# Neurexin-1 and Frontal Lobe White Matter: An Overlapping Intermediate Phenotype for Schizophrenia and Autism Spectrum Disorders

**DOI:** 10.1371/journal.pone.0020982

**Published:** 2011-06-08

**Authors:** Aristotle N. Voineskos, Tristram A. P. Lett, Jason P. Lerch, Arun K. Tiwari, Stephanie H. Ameis, Tarek K. Rajji, Daniel J. Müller, Benoit H. Mulsant, James L. Kennedy

**Affiliations:** 1 Centre for Addiction and Mental Health, University of Toronto, Toronto, Canada; 2 Hospital for Sick Children, University of Toronto, Toronto, Canada; Ecole Polytechnique Federale de Lausanne, Switzerland

## Abstract

**Background:**

Structural variation in the neurexin-1 (*NRXN1*) gene increases risk for both autism spectrum disorders (ASD) and schizophrenia. However, the manner in which *NRXN1* gene variation may be related to brain morphology to confer risk for ASD or schizophrenia is unknown.

**Method/Principal Findings:**

53 healthy individuals between 18–59 years of age were genotyped at 11 single nucleotide polymorphisms of the *NRXN1* gene. All subjects received structural MRI scans, which were processed to determine cortical gray and white matter lobar volumes, and volumes of striatal and thalamic structures. Each subject's sensorimotor function was also assessed. The general linear model was used to calculate the influence of genetic variation on neural and cognitive phenotypes. Finally, *in silico* analysis was conducted to assess potential functional relevance of any polymorphisms associated with brain measures. A polymorphism located in the 3′ untranslated region of *NRXN1* significantly influenced white matter volumes in whole brain and frontal lobes after correcting for total brain volume, age and multiple comparisons. Follow-up *in silico* analysis revealed that this SNP is a putative microRNA binding site that may be of functional significance in regulating *NRXN1* expression. This variant also influenced sensorimotor performance, a neurocognitive function impaired in both ASD and schizophrenia.

**Conclusions:**

Our findings demonstrate that the *NRXN1* gene, a vulnerability gene for SCZ and ASD, influences brain structure and cognitive function susceptible in both disorders. In conjunction with our *in silico* results, our findings provide evidence for a neural and cognitive susceptibility mechanism by which the *NRXN1* gene confers risk for both schizophrenia and ASD.

## Introduction

Autism Spectrum Disorders (ASDs) and schizophrenia are highly heritable disorders with genetic factors comprising the majority of the known risk [1]. Currently, the gene with the best evidence for shared susceptibility for schizophrenia and ASD is the Neurexin-1 (*NRXN1*) gene, one of the largest known human genes (1.1 Mb) with 24 exons, located on chromosome 2p16.3 [2]. The *NRXN1* gene encodes the neurexin-1α and neurexin-1β proteins that function as pre-synaptic neural adhesion molecules. Neurexin-1α is reported to interact with postsynaptic neuroligins (NLGNs) mediating GABAergic and glutamatergic synapse function [2]. It also has been reported to bind to leucine-rich repeat transmembrane protein (LRRTM2) [3], instructing presynaptic and mediating postsynaptic differentiation of glutamatergic synapses. Substantial evidence implicates deletions in the *NRXN1* gene in ASD [4]–[10] and schizophrenia [11]–[17]. *NRXN1* has also been associated with mental retardation [18], [19], nicotine dependence [20]–[22], alcoholism [23] and vertebral anomalies [24]. Therefore, it is apparent that disruptions of the *NRXN1* gene, especially deletions, confer risk to a range of neurodevelopmental phenotypes, including ASDs, schizophrenia, and mental retardation.

The results of neuroimaging studies suggest that schizophrenia and ASD patients also share neural vulnerability, most notably in the frontal lobe and in frontal lobe circuitry [25], [26]. Therefore, genes that confer susceptibility to both schizophrenia and ASD might contribute to altered brain structure and/or function common to both disorders. Although few studies have included both ASD and schizophrenia patients, overlapping findings between these illnesses occur most prominently in the frontal lobe and in fronto-striatal circuitry [25], [26]. Grey and white matter in ASD has been associated with increased cortical grey to white matter ratio and decreased volumes beyond childhood [27], [28]. Although both increases and decreases in grey and white matter volumes in ASD have been reported, white matter abnormalities in the frontal lobe remain some of the most consistent neuroimaging findings in ASD [29]–[36]. Thus, developmental abnormalities in white matter growth seems important in the etioneuropathology of ASD [37]. Structural MRI findings in schizophrenia populations are typically characterized by decreases in temporal and frontal lobe volumes, and some reductions in total brain volume and parietal volumes [38], [39]. Although findings have not always been consistent, a recent meta-analysis of 17 studies confirmed a frontal lobe white matter deficit in patients with schizophrenia [40]. Furthermore, cytoarchitectural alterations of the prefrontal cortex have been found in schizophrenia, and decreased thalamic volume and altered prefrontal-thalamic circuitry are common findings in this disorder [41]–[47]. Altogether, these findings suggest abnormalities of frontal, thalamic, and striatal structure that may be shared in the neuropathology of schizophrenia and ASD. Neurocognitively, sensorimotor deficits are shared by both disorders. Such deficits are typically apparent in ASD patients [48]. Cognitive assessment [49] and birth cohort studies [50] also identify impaired sensorimotor function in schizophrenia.

The intermediate phenotype approach permits us to examine how shared genetic underpinnings of these two disorders may confer risk in the brain [51]–[53]. Therefore, we used this approach to investigate 11 single nucleotide polymorphisms (SNPs) of the *NRXN1* gene lying within regions overlapped by numerous deletions implicated in ASD and schizophrenia, and their effects on brain morphometry in healthy individuals. Given that such deletions confer susceptibility to both schizophrenia and ASD, we hypothesized that *NRXN1* polymorphisms would confer an intermediate phenotype related to schizophrenia and ASD, via effects on neural structures and cognitive function altered in both disorders.

## Results

### Genotypes

**Figure 1:**
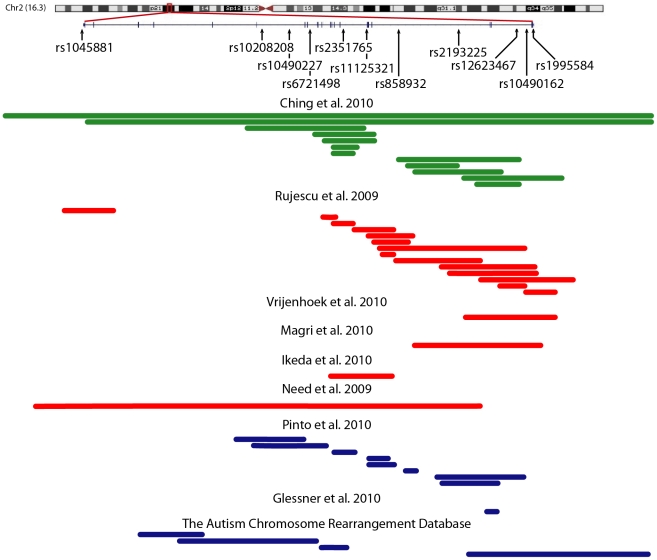
Reported Deletions in the Neurexin-1α gene. Figure contains the location of gene, markers, and reported deletion in: developmental disorders (green; Ching et al. [18]), schizophrenia (red; Rujescu et al. [13], Vrijenhoek et al. [14], Magri et al. [96], Ikeda et al. [15], Need et al. [12]), and autism spectrum disorders (blue; Pinto et al. [97], Glessner et al. [17]. The Autism Chromosome Rearrangement Database [6]). Figure adapted from the UCSC genome browser (GRCh37/hg19 assembly) [98].

Concordance for the 10% of re-genotyping of all 11 SNPs (Figure 1) was 100%. No SNP deviated significantly from Hardy-Weinberg equilibrium. Four SNPs (rs10208208, rs12623467, rs10490162, 10490227) were not included in further analysis since their minor allele frequency (MAF) was below 15% (Table S1). Furthermore, none of the SNPs was in linkage disequilibrium (LD) (not shown), and their MAF was similar to the Hapmap CEU population [54]. For rs1045881 since only one TT homozygote was in the sample, we combined T-allele carriers (T/T and T/C) and collectively analyzed in one cell. Post hoc independent t-tests of rs1045881 genotype (T-Carriers vs. C/C) revealed no significant differences in any demographics measured (Table S2).

**Figure 2:**
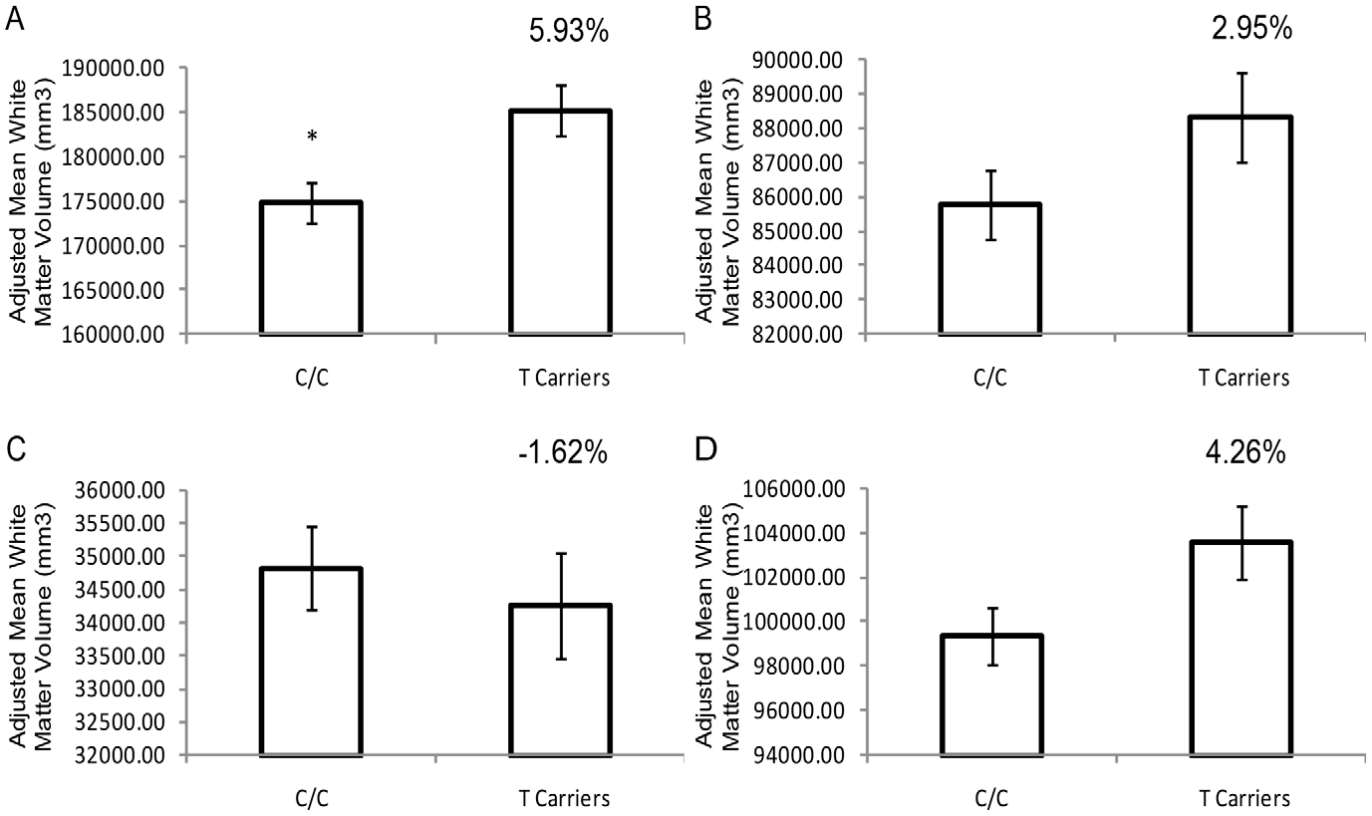
The effect of rs1045881 on combined hemispheric volume of brain regions with total brain volume (TBV) and age as covariates. Brain regions: (A) Frontal Lobe, (B) Temporal Lobe, (C) Occipital Lobe, and (D) Parietal Lobe. Frontal lobe white matter volume was significantly greater in T allele carriers (T/T +T/C) (ANCOVA F_1,52_ = 8.197, p = 0.006), while other regions are non-significant after correcting for multiple comparisons. Covariates appearing in the model are evaluated at the following values: TBV = 1364768.17, Age = 39.04, (*) denotes significance of P<0.0125. Error bars represent +/− standard error of the marginal means and percentages reflect the percent change in each brain region.

For lobar gray matter volumes, no genotype by brain region interactions or main effects of genotype were found following repeated measure ANCOVAs conducted for each of the seven SNPs with MAF>15%, with age and total brain volume as covariates. Therefore, no follow-up analysis was performed. When examining white matter volumes, we found that for each lobe, a minimum of 85% of the variance in one hemisphere was explained by the white matter volume of the other hemisphere (P<0.001, R^2^_(Pearson)_>0.85); therefore, we combined lobar white matter volumes across hemispheres. For lobar white matter volumes, a genotype by white matter lobe volume interaction was found following repeated measures ANCOVA, at the rs1045881 (F_2.25_ = 5.498, p = 0.004) and rs858932 (F_4.56_ = 3.802, p = 0.004) polymorphisms (Bonferroni corrected alpha of 0.0071). We did not observe significant white matter region volume by genotype interactions in any other *NRXN1* variants examined. The results for the rs1045881 and rs858932 SNPs were followed up using separate ANCOVAs for white matter volume at each lobe. The rs1045881 variant was significantly associated with frontal lobe white matter volume (Bonferroni corrected alpha = 0.0125 for four brain regions): F_1,49_ = 8.231, p = 0.006; (Figure 2), where ‘CC’ homozygotes demonstrated reduced frontal white matter volumes compared to ‘T’ allele carriers. Consistent with the direction of effect in frontal lobe, the rs1045881 was nominally associated (as it did not survive Bonferroni correction) with change in parietal lobe white matter volume (F_1,49_ = 4.089, p = 0.049). No association of this SNP with temporal or occipital lobe white matter volume was observed.

The follow-up ANCOVA examining rs858932 genotype also predicted frontal lobe white matter volume (F_2,51_ = 5.472, p = 0.007), where ‘GG’ individuals had lower frontal lobe white matter volume and nominal association in the parietal lobe also occurred in the same direction, but did not survive Bonferroni correction (F_48,2_ = 3.719, p = 0.032; Figure S1). Frontal lobe white matter volumes were also associated at the allelic level: both the ‘C’ allele of rs1045881 (χ^2^ = 7.184, p = 0.0074) and the ‘G’ allele of rs858932 (χ^2^ = 4.121, p = 0.0432) predicted lower frontal white matter volume (Table S3). Similar results were shown in the haplotype analysis (p_(Global)_<0.001; Table S4).

**Figure 3:**
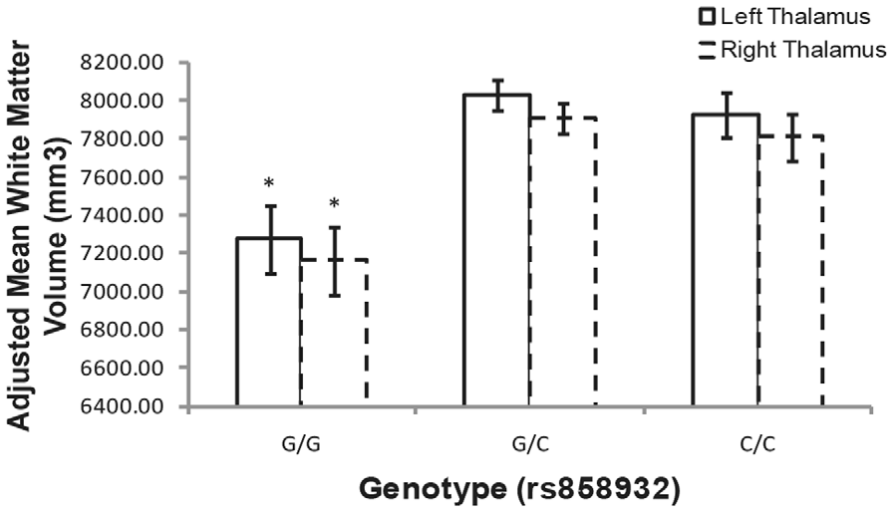
The effect of rs858932 on right and left thalamic volume with TBV and age as covariates. There are approximately 10% and 9% percent differences between the G/G to G/C and G/G and C/C genotypes for both thalamic hemispheres, respectively. Covariates appearing in the model are evaluated at the following values: TBV = 1364768.17, Age = 39.04, (*) denotes significance of P<0.0063. Error bars represent +/− standard error of the marginal means.

Repeated measures analysis for striatal and thalamic structures revealed a significant volume by region interaction for the rs858932 SNP only (F_14,336_ = 3.4, p<0.001; Greenhouse-Geiser correction: F_4,99_ = 3.4, p = 0.01). Follow-up ANCOVAs at left and right caudate, putamen, globus pallidus, and thalamus revealed that this interaction was driven by the influence of the rs858932 SNP on thalamic volumes only: for left thalamus (F_2,48_ = 8.9, p = 0.001), and for right thalamus (F_2,48_ = 7.3, p = 0.002), significant at the Bonferroni corrected alpha for eight comparisons (alpha = 0.0063, Figure 3). Here, ‘GG’ individuals had significantly lower thalamic volumes compared to ‘T’ allele carriers. No significant effects were observed at caudate, putamen, or globus pallidus.

### Cognitive

Repeated measures ANCOVA showed a main effect of the rs1045881 SNP on sensorimotor function (F_1,49_ = 4.8, p = 0.03). The ‘C/C’ homozygotes had reduced finger tapping scores compared to ‘T’ allele carriers, consistent with the directional effect on white matter volumes. No association was observed for the rs858932 SNP (F_1,48_ = 0.4, p = 0.67). No task by genotype interaction was observed for either polymorphism.

Frontal lobe white matter volume was highly correlated with finger tapping (FT) score even after accounting for age effects (Dominant Hand: R^2^ = 0.404, p = 0.003; Non-Dominant Hand: R^2^ = 0.469, p = 0.001).

### *In silico* analysis

The rs1045881 SNP is located in the 3′UTR of Neurexin-1. *In silico* prediction by miRBase analysis revealed the presence of the C-allele creates a binding site for the microRNA hsa-miR-1274a and hsa-miR-339-5p. Furthermore, alteration in exon splicing enhancer and other motifs were observed. The rs858932 SNP was not sufficiently near any splice site (i.e. intron/exon border) for *in silico* prediction.

## Discussion

We found that genetic variation in the 3′untranslated region of the *NRXN1* gene predicted an intermediate risk phenotype in healthy individuals relevant to schizophrenia and ASD. Our primary finding at the rs1045881 SNP in the 3′UTR of Neurexin1 demonstrated that the ‘C’ allele predicts reduced frontal white matter volume and sensorimotor function. Furthermore, our *in silico analysis* suggested presence of the same ‘C’ allele predicted microRNA binding, thus providing a potential mechanism for this allele's effects on brain structure and cognitive function. The gene variants that influenced brain morphology in our study are located in the regions of *NRXN1* susceptible to deletion in schizophrenia and ASD. The effects of these genetic variants localized to brain structure and cognitive function that demonstrate overlapping susceptibility in both schizophrenia and ASD, namely frontal lobe white matter abnormalities, as shown in recent meta-analyses [40], [55] and sensorimotor function [56]–[59]. To our knowledge, this work provides the first evidence *in vivo* of how variation in the *NRXN1* gene may confer a potential neural risk mechanism for schizophrenia and ASD.

Schizophrenia and ASD patients share sensorimotor deficits and soft neurological signs [60]. Such shared deficits are almost certainly neurodevelopmental in nature, as in ASD they present at a very early age, and when present in schizophrenia, they are often present before illness onset. White matter, likely through myelination, plays a key role in ensuring appropriate sensorimotor development, and motor tasks and motor speed are tightly correlated with white matter indices on MRI [61], [62]. Our finding correlating white matter volumes with sensorimotor performance is consistent with previous investigations [33], [63], [64]. Moreover, the same *NRXN1* allele that predicted microRNA binding (and thus presumably increased enzymatic breakdown of *NRXN1* mRNA and reduced *NRXN1* translation) also correlates with reduced white matter volumes and altered sensorimotor function.

Our second finding was that the intronic rs858932 SNP, also located in a deletion site [13], [14], [18], similarly influenced frontal lobe white matter volume, but also prominently influenced left and right thalamic volumes. We consider this finding more preliminary due to the lower minor allele frequency at this variant in our sample. Nevertheless, association of this variant with thalamic volumes is consistent with overlapping neural vulnerability for ASD and schizophrenia as well [39], [65], and suggests that the *NRXN1* gene may influence thalamocortical circuitry that is vulnerable in both disorders.

Little is known about how specific types of deletions within the *NRXN1* gene may relate to a given neuropsychiatric phenotype. Our *in silico* analysis demonstrated the 3′UTR SNP as a putative microRNA binding site for hsa-miR-339 and hsa-miR-1274, thus suggesting a functional role for this region of the gene that may relate to mRNA expression of *NRXN1*. This is interesting since expression of miR-339 microRNA has been reported to be dysregulated in the cortex of psychotic patients [66]. Reduced *NRXN1* mRNA may influence white matter alterations by concomitant reductions in binding to the *NRXN1* binding partner, LRRTM2, which mediates postsynaptic differentiation of glutamatergic synapses [3], [67], [68]. Glutamatergic dysfunction is well established in schizophrenia [69]; further, *NXRN1* expression is induced by AMPA receptors, and mediates recruitment of NMDA receptors, a hallmark of synapse maturation [70]. Glutamatergic dysfunction can also lead to white matter abnormalities. Oligodendrocytes possess glutamatergic receptors (both AMPA and NMDA), and are highly sensitive to any form of stress or toxicity [71]. Therefore, *NRXN1* may influence frontal white matter in schizophrenia and ASD through disrupted interaction with its glutamatergically-related binding partners, or possibly via direct glutamatergic involvement as the *NRXN1* knock out mouse demonstrates decreased excitatory synaptic strength and decreased prepulse inhibition [72].

Recent imaging-genetics studies [73], [74] have implicated a neurexin superfamily member, the *contactin-associated protein-like 2* (*CNTNAP2*) gene in brain structure and function providing evidence for neural susceptibility patterns relevant to ASD. These studies demonstrated volumetric reductions for *CNTNAP2* risk allele carriers particularly in frontal lobe [73], [74] and also showed altered frontal connectivity. One of these two studies [74] demonstrated strong effects with sample sizes smaller than ours. Our findings, in conjunction with the recent imaging-genetics findings of *CNTNAP2* demonstrate the value of examining common variants within known ASD risk genes to understand neural susceptibility mechanisms conferred by these risk genes. The ‘added-value’ of this approach lies in the neural localization of gene effects, providing information regarding how the genes may confer brain risk patterns for these disorders.

There are several limitations in our study that should be considered. First, we imposed a dominant model by combining genotypic groups C/T and T/T at rs1045881; however concern regarding this model can be mitigated by our findings that allelic association analysis supported such a model. Second, one could argue that our finding may constitute a ‘winner's curse’, and therefore we would encourage replication efforts. A third limitation of our study is that while there was a clear effect of this putative risk variant on frontal lobe white matter volume, in a direction consistent with cognitive function findings and *in silico* prediction, various MRI studies have reported either reductions or increases in frontal lobe white matter for both populations. Finally, given that we measured gray and white matter volumes for cortical lobar structures, we were somewhat limited in obtaining more localized regional specificity for effects of *NRXN1* variation. More detailed parcellation, white matter voxel-based morphometry, or other white matter imaging techniques such as diffusion tensor imaging, magnetization transfer imaging, or T2 techniques should help clarify further the manner in which *NRXN1* influences frontal white matter.

In summary, we found that variants within the *NRXN1* gene influence brain morphometry with a susceptibility pattern relevant to both schizophrenia and ASD. This finding is consistent with the fact that *NRXN1* is a vulnerability gene for both disorders. In addition to reporting that the rs1045881 gene variant is associated with frontal white matter volume and sensorimotor performance, we provide a putative mechanistic explanation for its effects in the brain. Taken together, our findings provide evidence that genetic variation in *NRXN1*, a risk gene for schizophrenia and ASD, may confer neural and cognitive susceptibility common to both disorders.

## Materials and Methods

### Participants

Fifty-three healthy volunteers (15 women, 38 men) (Table 1) met the following eligibility criteria: age between 18 and 59; right handedness; absence of any history of a mental disorder, current substance abuse or a history of substance dependence, positive urine toxicology, history of head trauma with loss of consciousness, seizure, or another neurological disorder; no first degree relative with a history of psychotic mental disorder. All participants were assessed with the Edinburgh handedness inventory [75] for handedness, Wechsler Test for Adult Reading (WTAR) for IQ, and Hollingshead index for socio-economic status [76]. They were interviewed by a psychiatrist, and completed the Structured Clinical Interview for DSM-IV Disorders [77]. They also completed a urine toxicology screen. The study was approved by the Research Ethics Board of the Centre for Addiction and Mental Health (Toronto, Canada) and all participants provided informed, written consent.

**Table 1:** Demographic Characteristics.

	Mean± St. Dev.	Range
Age	39.0±13.1	19–59
Education (years)	15.6±2.0	12–20
IQ (WTAR)	118.2±7.7	92–127
Socioeconomic Status^a^	50.0±9.8	27–66

WTAR, Wechsler Test of Adult Reading.

a Composed of four factors are education, occupation, sex, and marital status.

### Neuroimaging

High resolution magnetic resonance images were acquired as part of a multi-modal imaging protocol using an eight-channel head coil on a 1.5 Tesla GE Echospeed system (General Electric Medical Systems, Milwaukee, WI), which permits maximum gradient amplitudes of 40 mT/m. Axial inversion recovery prepared spoiled gradient recall images were acquired: echo time (TE) = 5.3, repetition time (TR) = 12.3, time to inversion (TI) = 300, flip angle = 20, number of excitations (NEX) = 1 (124 contiguous images, 1.5 mm thickness).

### Image Processing

Each subject's T1 image was submitted to the CIVET pipeline (version 1.1.7) developed at the Montreal Neurological Institute [78]. The processing steps included registration to the symmetric ICBM 152 template [79] with a 12-parameter linear transformation [80], correction for inhomogeneity artifact [81], skull stripping [82], tissue classification into white and grey matter, cerebrospinal fluid and background [83], [84] and neuroanatomical segmentation using ANIMAL [85]. Total volumes for each cortical lobe and subcortical structures were estimated for each individuals by non-linearly warping each T1 image towards a segmented atlas [86]. Volume (mL) was extracted from each of these regions using the RMINC package (version 0.4) for reading and analyzing MINC2 output files. Total gray matter, white matter, and CSF volumes were calculated, along with lobar cortical gray and white matter volumes (i.e., left and right frontal, temporal, parietal, occipital), along with volumes of subcortical structures related to the fronto-striato-thalamic loop implicated in both schizophrenia and ASD including left and right caudate, putamen, globus pallidus, and thalamus.

### Genetics

Genomic data was extracted from ethylenediametetraaecidic acid (EDTA) anticoagulated venous blood according to standard procedures. Eleven SNPs were genotyped on an Applied Biosystems ABI 7500 Real-Time PCR system, using Taqman 5′ nuclease assay. Genotyping accuracy was assessed by running 10% of the sample in duplicate. Eleven SNPs were selected across the *NRXN1* gene (NC_000002.11). Each marker is located in reported regions within which multiple rare deletions associated with ASD and schizophrenia (Figure 1, Table S5).

The program Haploview 4.2 [87] was used to determine pair-wise LD between all SNPs with blocks determined by the Gabriel et al. method [88]. Haploview 4.2 was also used to determine whether SNPs were in Hardy Weinberg equilibrium.

### Cognitive assessment

Fifty-two of the study participants completed cognitive testing that included the finger-tapping test [89]–[91]. Although cognitive deficits in ASD are not as well-characterized as those in schizophrenia, sensorimotor function is disrupted in both disorders [92]–[95]. Therefore, we used the finger-tapping test to assess sensorimotor function.

### Statistical Analysis

Statistical analysis was performed using SPSS for Windows 15.0. To test for effects of *NRXN1* genotype on brain morphometry, three separate repeated measures ANCOVA (for cortical lobar gray matter, cortical lobar white matter, and subcortical structures) were performed with genotype as the between group factor, brain region volume as the within group factor, and age and total brain volume (TBV) as covariates. To ensure adequate power, only markers with a minor allele frequency (MAF) greater than 15% were tested. We used a Bonferroni correction based on multiple comparisons of 7 SNPs to determine significance (alpha = 0.0071). Where the repeated measures ANCOVA revealed a significant volume by genotype interaction, follow-up ANCOVAs were performed and Bonferroni correction applied. When significant effect of a genotype on brain volume was found, UNPHASED 3.1 was then used to examine allelic association with brain phenotypes. Haplotype quantitative analysis of frontal lobe white matter volume and the rs1045881 and rs858932 *NRXN1* variants were calculated using haplotype score (Methods S1). Finally, for those genotypes that significantly predicted brain measures, repeated measures ANCOVA for sensorimotor function was performed (dominant and nondominant finger-tapping scores as within group measures) with age as covariate. For any gene variant that predicted both brain measures and cognitive performance, the relationship between that brain measure and cognitive performance was examined using a linear regression model, accounting for age effects.

### *In Silico* Analysis

In order to enhance the understanding of the biological meaningfulness of the genetic associations, we used *in silico* methods to predict potential function of the SNPs investigated in this study. Depending on their location, SNPs were assessed for alteration in transcription factor binding using MatInspector (Genomatix; promoter and intron 1). Presence of splicing enhancers, repressors or intronic regulatory elements (intronic and exonic, synonymous and nonsynonymous SNPs) were determined using F-SNP (http://compbio.cs.queensu.ca/F-SNP) and Human Splicing Finder (http://www.umd.be/HSF/). 3′UTR SNPs were also assessed for alteration in microRNA binding sites (http://www.targetscan.org/).

## Supporting Information

Figure S1 **The effect of rs858932 on combined hemispheric volume of brain regions with TBV and age as covariates.** Brain regions: (A) Frontal Lobe, (B) Temporal Lobe, (C) Occipital Lobe, and (D) Parietal Lobe. Frontal and parietal lobe white matter volumes were significantly greater in G allele carriers (T/T +T/C) (ANCOVA F_2,52_ = 7.074, p = 0.002; ANCOVA F_2,52_ = 5.724, p = 0.006). Other region are non-significant after correcting for multiple comparisons. Covariates appearing in the model are evaluated at the following values: TBV = 1364768.17, Age = 39.04, (*) denotes significance of P<0.0125. Error bars represent +/− standard error of the marginal means and percentages reflect the percent change in each brain region.
(TIFF)

Table S1 **Locations and Minor Allele Frequency in Toronto and Hapmap (CEU) Samples.**
(DOC)

Table S2 **T-test between rs1045881 T-Carriers Vs C/C and Demographics.**
(DOC)

Table S3 **Chi-squared Tests of Region by Genotype or Allele Interactions of rs1045881 and rs858932.** Analysis was performed by Unphased 3.1 with total brain volume and age as confounding factors.
(DOC)

Table S4 **Haplotype Association between Frontal Lobe White Matter and rs1045881 (T/C) and rs858932 (G/C).**
(DOC)

Table S5 **Reported deletions within NRXN1 in Developmental Disorders, Schizophrenia and Autism Spectrum Disorders.**
(DOC)

Methods S1 **Haplotype Analysis.**
(DOC)
