# Supplementary figures and images for: Neurexin-1 and Frontal Lobe White Matter: An Overlapping Intermediate Phenotype for Schizophrenia and Autism Spectrum Disorders
Source: PLoS One. 2011 Jun 8;6(6):e20982. doi: 10.1371/journal.pone.0020982 (PMC3110800; doi:10.1371/journal.pone.0020982)

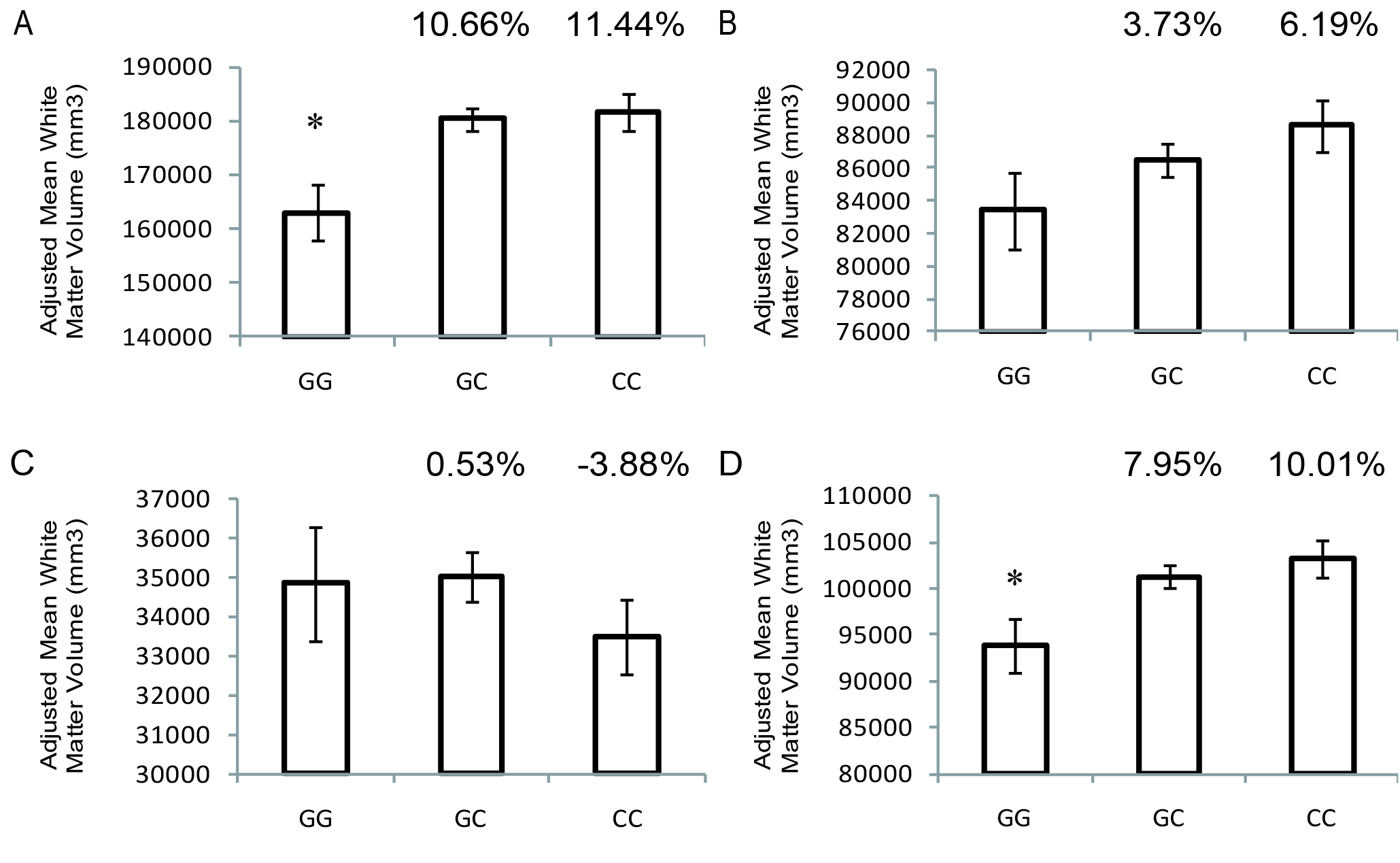

Supplement: Figure S1 — The effect of rs858932 on combined hemispheric volume of brain regions with TBV and age as covariates. Brain regions: (A) Frontal Lobe, (B) Temporal Lobe, (C) Occipital Lobe, and (D) Parietal Lobe. Frontal and parietal lobe white matter volumes were significantly greater in G allele carriers (T/T +T/C) (ANCOVA F2,52 = 7.074, p = 0.002; ANCOVA F2,52 = 5.724, p = 0.006). Other region are non-significant after correcting for multiple comparisons. Covariates appearing in the model are evaluated at the following values: TBV = 1364768.17, Age = 39.04, (*) denotes significance of P<0.0125. Error bars represent +/− standard error of the marginal means and percentages reflect the percent change in each brain region. (TIFF) [file pone.0020982.s001.tif]
