# Supplementary material for: Neurexin-1 and Frontal Lobe White Matter: An Overlapping Intermediate Phenotype for Schizophrenia and Autism Spectrum Disorders
Source: PLoS One. 2011 Jun 8;6(6):e20982. doi: 10.1371/journal.pone.0020982 (PMC3110800; doi:10.1371/journal.pone.0020982)
Supplement: Table S1 — Locations and Minor Allele Frequency in Toronto and Hapmap (CEU) Samples. (DOC) [file pone.0020982.s002.doc]

**Table S1.** **Locations and Minor Allele Frequency in Toronto and Hapmap (CEU) Samples.**

| Marker | Position^a^ | Alleles | Strand | Location | MAF | MAF (CEU)^b^ |
| --- | --- | --- | --- | --- | --- | --- |
| rs1995584 | 51263149 | A/G | + | 5’ Pro | A: 0.446 | A: 0.446 |
| rs10490162 | 51247657 | A/G | - | Intron | G: 0.054 | G: 0.102 |
| rs12623467 | 51225089 | C/T | + | Intron | T: 0.027 | T: 0.050 |
| rs2193225 | 51079482 | A/G | - | Intron | A: 0.446 | A: 0.496 |
| rs858932 | 50930063 | C/G | - | Intron | G: 0.420 | G: 0.442 |
| rs11125321 | 50852016 | A/G | + | Intron | G: 0.375 | G: 0.403 |
| rs2351765 | 50793780 | A/C | + | Intron | A: 0.312 | A: 0.274 |
| rs6721498 | 50713011 | A/G | + | Intron | G: 0.464 | G: 0.492 |
| rs10490227 | 50659515 | A/G | - | Intron | A: 0.143 | A: 0.093 |
| rs10208208 | 50593914 | G/T | + | Intron | T: 0.000 | T: 0.175 |
| rs1045881 | 50148972 | A/G | - | 3’UTR | T: 0.205 | T: 0.129 |

MAF = Minor Allele Frequency; ^a^According to dbSNP build 131; ^b^Hapmap CEU Sample
