# Supplementary material for: Neurexin-1 and Frontal Lobe White Matter: An Overlapping Intermediate Phenotype for Schizophrenia and Autism Spectrum Disorders
Source: PLoS One. 2011 Jun 8;6(6):e20982. doi: 10.1371/journal.pone.0020982 (PMC3110800; doi:10.1371/journal.pone.0020982)
Supplement: Table S2 — T-test between rs1045881 T-Carriers Vs C/C and Demographics. (DOC) [file pone.0020982.s003.doc]

**Table S2. T-test between rs1045881 T-Carriers Vs C/C and Demographics.**

|  | Genotype | N | Mean | Std. Dev. | t | df | *p*-value |
| --- | --- | --- | --- | --- | --- | --- | --- |
| Age | TT + TC | 20 | 38.55 | 12.959 | -0.209 | 51 | 0.836 |
|  | CC | 33 | 39.33 | 13.411 |  |  |  |
| Education | TT + TC | 18 | 15.89 | 1.568 | 0.893 | 48 | 0.377 |
|  | CC | 32 | 15.38 | 2.136 |  |  |  |
| WTAR | TT + TC | 19 | 118.53 | 6.230 | 0.370 | 50 | 0.713 |
|  | CC | 33 | 117.70 | 8.527 |  |  |  |
| MMSE | TT + TC | 20 | 29.60 | 0.821 | 1.202 | 50 | 0.235 |
|  | CC | 32 | 29.28 | 0.991 |  |  |  |
| CIRSG | TT + TC | 18 | 1.28 | 1.841 | -0.232 | 48 | 0.817 |
|  | CC | 32 | 1.41 | 1.898 |  |  |  |
| SE Status ^a^ | TT + TC | 17 | 51.12 | 8.667 | 0.736 | 43 | 0.466 |
|  | CC | 28 | 48.89 | 10.457 |  |  |  |

WTAR, Wechsler Test of Adult Reading; MMSE, Mini Mental State Examination; CIRS-G, Cumulative Illness Rating Scale - Geriatrics; SE, Socioeconomic status. ^a^Composed of four factors: education, occupation, sex, and marital status.
