# Supplementary material for: Neurexin-1 and Frontal Lobe White Matter: An Overlapping Intermediate Phenotype for Schizophrenia and Autism Spectrum Disorders
Source: PLoS One. 2011 Jun 8;6(6):e20982. doi: 10.1371/journal.pone.0020982 (PMC3110800; doi:10.1371/journal.pone.0020982)
Supplement: Table S3 — Chi-squared Tests of Region by Genotype or Allele Interactions of rs1045881 and rs858932. Analysis was performed by Unphased 3.1 with total brain volume and age as confounding factors. (DOC) [file pone.0020982.s004.doc]

**Table S3.** **Chi-squared Tests of Region by Genotype or Allele Interactions of rs1045881 and rs858932.** Analysis was performed by Unphased 3.1 with total brain volume and age as confounding factors.

|  | **rs1045881** | | | | **rs858932** | | | |
| --- | --- | --- | --- | --- | --- | --- | --- | --- |
|  | Allelic  (C vs T)^a^ | | Genotypic  (T-Carriers vs C/C)^b^ | | Allelic  (G vs C)^c^ | | Genotypic  (G/G vs G/C vs C/C)^d^ | |
| Region | χ^2^ | *p*-value | χ^2^ | *p*-value | χ^2^ | *p*-value | χ^2^ | *p*-value |
| Frontal Lobe | 7.1840 | **0.0074** | 8.4151 | **0.0037** | 4.1213 | 0.0423 | 10.0033 | **0.0067** |
| Temporal Lobe | 1.8624 | 0.1723 | 2.4474 | 0.1177 | 2.4568 | 0.1170 | 3.9295 | 0.1402 |
| Occipital Lobe | 0.4720 | 0.4921 | 0.1402 | 0.5639 | 0.8973 | 0.3435 | 1.8958 | 0.3876 |
| Parietal Lobe | 2.8906 | 0.0891 | 4.2641 | 0.0389 | 4.0856 | 0.0432 | 8.5304 | 0.0140 |

Bold values are significant after Bonferroni correction alpha for multiple comparisons (α=0.0125). ^a^(T:C=21:85); ^b^(T-carriers:C/C = 20:33); ^c^(G:C = 45:61); ^d^(G/G:G/C:C/C=6:33:14).
