# Supplementary material for: Neurexin-1 and Frontal Lobe White Matter: An Overlapping Intermediate Phenotype for Schizophrenia and Autism Spectrum Disorders
Source: PLoS One. 2011 Jun 8;6(6):e20982. doi: 10.1371/journal.pone.0020982 (PMC3110800; doi:10.1371/journal.pone.0020982)
Supplement: Table S4 — Haplotype Association between Frontal Lobe White Matter and rs1045881 (T/C) and rs858932 (G/C). (DOC) [file pone.0020982.s005.doc]

**Table S4.** **Haplotype Association between Frontal Lobe White Matter and rs1045881 (T/C) and rs858932 (G/C).**

^a^ Age and Total Brain Volume are covariates.

| Haplotype Test of Overall Association with Frontal Lobe White Matter^a^ | | | |
| --- | --- | --- | --- |
| Global Score Statistic | df | *p*-value |  |
| 21.92665 | 3 | 7 x 10^-5^ |  |
| Estimates of Haplotype Main Effects^a^ | | | |
| Haplotype | Haplotype Score | Freq | *p*-value |
| T/G | 2.12585 | 0.09992 | 0.03352 |
| T/C | 2.49622 | 0.09819 | 0.01255 |
| C/G | -3.9051 | 0.32461 | 9 x 10^-5^ |
| C/C | 1.10893 | 0.47728 | 0.26746 |
