# Supplementary material for: Neurexin-1 and Frontal Lobe White Matter: An Overlapping Intermediate Phenotype for Schizophrenia and Autism Spectrum Disorders
Source: PLoS One. 2011 Jun 8;6(6):e20982. doi: 10.1371/journal.pone.0020982 (PMC3110800; doi:10.1371/journal.pone.0020982)
Supplement: Table S5 — Reported deletions within NRXN1 in Developmental Disorders, Schizophrenia and Autism Spectrum Disorders. (DOC) [file pone.0020982.s006.doc]

**Table S5**. **Reported deletions within NRXN1 in Developmental Disorders, Schizophrenia and Autism Spectrum Disorders.**

| Start | Stop | Diagnosis |
| --- | --- | --- |
| Ching et al. (2010) [1] | | |
| 46938685 | 52015885 | Moderate mental retardation |
| 50128256 | 54050713 | Global developmental delays, suspected autism |
| 50897002 | 51212385 | Gross motor delay, hypotonia |
| 50936914 | 51167934 | PDD-NOS, hypotonia |
| 50920082 | 51059469 | VACTERL |
| 51059410 | 51316396 | PDD-NOS, motor coordination delays |
| 51090504 | 51212385 | Autism, moderate mental retardation |
| 50522892 | 50827767 | Mild mental retardation |
| 50689280 | 50853329 | Language delay, prenatal substance exposure |
| 50714297 | 50853329 | PDD-NOS |
| 50735499 | 50811018 | Hypotonia, muscle weakness, large birth weight |
| 50735499 | 50801233 | Poor weight gain, mild craniofacial dysmorphism |
| Rujescu et al. (2009) [2] | | |
| 50856110 | 50900862 | Schizophrenia |
| 50890216 | 51116653 | Schizophrenia, social contact problems in childhood |
| 51147600 | 51225851 | Schizophrenia |
| 50071499 | 50208992 | Schizophrenia, chronic, positive symptoms, low IQ (82),  low educational level |
| 50822312 | 50948557 | Schizophrenia, chronic, negative symptoms, episode  of aggression |
| 51101161 | 51344213 | Schizophrenia, alcoholism |
| 50735657 | 50800548 | Schizophrenia |
| 50786446 | 50900862 | Schizophrenia |
| 51002576 | 51250922 | Schizophrenia, episodic with partial remission and negative symptoms |
| 51024962 | 51251873 | Schizophrenia |
| 51211406 | 51299436 | Schizophrenia, myoclonic seizures in the right shoulder |
| 50711199 | 50756435 | Schizophrenia |
| 50836690 | 50936258 | Schizophrenia |
| 50850456 | 51225851 | Schizophrenia, mental retardation (mild), Tardive dyskinesia |
| Vrijenhoek et al. (2008) [3] | | |
| 51063670 | 51300517 | Schizophrenia |
| Magri et al. (2010) [4] | | |
| 50952424 | 51280162 | Schizophrenia |
| Ikeda et al. (2010) [5] | | |
| 50743926 | 50911879 | Schizophrenia |
| Need et al. (2009) [6] | | |
| 49999148 | 51113178 | Schizophrenia |
| Pinto et al. (2010) [7] | | |
| 50912249 | 50955087 | Autism Spectrum Disorder |
| 50493827 | 50677835 | Autism Spectrum Disorder |
| 50539877 | 50730546 | Autism Spectrum Disorder |
| 50990306 | 51222043 | Autism Spectrum Disorder |
| 51002576 | 51157742 | Autism Spectrum Disorder |
| 50735657 | 50804497 | Autism Spectrum Disorder |
| 50822312 | 50886363 | Autism Spectrum Disorder |
| 50822312 | 50900862 | Autism Spectrum Disorder |
| Glessner et al. (2009) [8] | | |
| 51120644 | 51147600 | Autism Spectrum Disorder |
| The Autism Chromosome Rearrangement Database [9] | | |
| 50371853 | 50727153 | Autism Spectrum Disorder |
| 50722055 | 50801053 | Autism Spectrum Disorder |
| 50273117 | 50443987 | Autism Spectrum Disorder |
| 51086655 | 59969199 | Autism Spectrum Disorder |
| 51759493 | 52031003 | Autism Spectrum Disorder |

**References**

1. Ching MSL, Shen Y, Tan W-H, Jeste SS, Morrow EM, et al. (2010) Deletions of NRXN1 (neurexin-1) predispose to a wide spectrum of developmental disorders. American Journal of Medical Genetics Part B, Neuropsychiatric Genetics: The Official Publication of the International Society of Psychiatric Genetics 153B: 937-947.

2. Rujescu D, Ingason A, Cichon S, Pietiläinen OPH, Barnes MR, et al. (2009) Disruption of the neurexin 1 gene is associated with schizophrenia. Human Molecular Genetics 18: 988-996.

3. Vrijenhoek T, Buizer-Voskamp JE, van der Stelt I, Strengman E, Sabatti C, et al. (2008) Recurrent CNVs disrupt three candidate genes in schizophrenia patients. American Journal of Human Genetics 83: 504-510.

4. Magri C, Sacchetti E, Traversa M, Valsecchi P, Gardella R, et al. (2010) New copy number variations in schizophrenia. PloS One 5: e13422.

5. Ikeda M, Aleksic B, Kirov G, Kinoshita Y, Yamanouchi Y, et al. (2010) Copy number variation in schizophrenia in the Japanese population. Biological Psychiatry 67: 283-286

6. Need AC, Ge D, Weale ME, Maia J, Feng S, et al. (2009) A genome-wide investigation of SNPs and CNVs in schizophrenia. PLoS Genetics 5: e1000373.

7. Pinto D, Pagnamenta AT, Klei L, Anney R, Merico D, et al. (2010) Functional impact of global rare copy number variation in autism spectrum disorders. Nature 466: 368-372.

8. Glessner JT, Wang K, Cai G, Korvatska O, Kim CE, et al. (2009) Autism genome-wide copy number variation reveals ubiquitin and neuronal genes. Nature 459: 569-573.

9. Marshall CR, Noor A, Vincent JB, Lionel AC, Feuk L, et al. (2008) Structural variation of chromosomes in autism spectrum disorder. American Journal of Human Genetics 82: 477-488.
