# Supplementary material for: Neurexin-1 and Frontal Lobe White Matter: An Overlapping Intermediate Phenotype for Schizophrenia and Autism Spectrum Disorders
Source: PLoS One. 2011 Jun 8;6(6):e20982. doi: 10.1371/journal.pone.0020982 (PMC3110800; doi:10.1371/journal.pone.0020982)
Supplement: Methods S1 — Haplotype Analysis. (DOC) [file pone.0020982.s007.doc]

**Methods S1. Haplotype Analysis**

Haplotype quantitative analysis of frontal lobe white matter volume and the rs1045881 and rs858932 *NRXN1* variants were calculated using haplotype score algorithm in haplostats in the R programming language (<http://mayoresearch.mayo.edu/mayo/research/schaid_lab/software.cfm>). Schaid et al.[1] developed a score statistic that can test the associations between haplotypes and a wide variety of traits, including binary, ordinal, quantitative, and Poisson. This method also allows for adjustment for non-genetic covariates. In our analysis, we used haplo.score to compute the global score statistic (that tests the significance of association of all haplotypes) and haplotype specific statistic (that compares each haplotype with selected common haplotypes). Our dependent variable was frontal lobe white matter volume. Our covariates were the TBV and age of the subjects. All haplotypes with a frequency less than 5% were dropped from the score test.

**References**

1. Schaid DJ, Rowland CM, Tines DE, Jacobson RM, Poland GA (2002) Score tests for association between traits and haplotypes when linkage phase is ambiguous. American Journal of Human Genetics 70: 425-434.
